# Preimplantation genetic testing for aneuploidy: A review of published blastocyst reanalysis concordance data

**DOI:** 10.1002/pd.5828

**Published:** 2020-10-04

**Authors:** Diego Marin, Jia Xu, Nathan R. Treff

**Affiliations:** ^1^ Genomic Prediction North Brunswick New Jersey USA; ^2^ Robert Wood Johnson Medical School, Department of Obstetrics, Gynecology, and Reproductive Sciences Rutgers University New Brunswick New Jersey USA

## Abstract

Preimplantation genetic testing for aneuploidy (PGT‐A) reduces miscarriage risk, increases the success of IVF, shortens time to pregnancy, and reduces multiple gestation rates without compromising outcomes. The progression of PGT‐A has included common application of next‐generation sequencing (NGS) from single nucleotide polymorphism microarray, quantitative real‐time PCR, and array comparative hybridization platforms of analysis. Additional putative advances in PGT‐A capability include classifying embryos as mosaic and predicting the presence of segmental imbalance. A critical component in the process of technical validation of these advancements involves evaluation of concordance between reanalysis results and initial testing results. While many independent studies have investigated the concordance of results obtained from the remaining embryo with the original PGT‐A diagnosis, compilation and systematic analysis of published data has not been performed. Here, we review results from 26 primary research articles describing concordance in 1271 human blastocysts from 2260 pairwise comparisons. Results illustrate significantly higher discordance from PGT‐A methods which utilize NGS and include prediction of mosaicism or segmental imbalance. These results suggest caution when considering new iterations PGT‐A.

**What's already known about this topic?**

- Several independent studies evaluating concordance between a clinical blastocyst trophectoderm biopsy and reanalysis of the remaining embryo have been published.

**What does this study add?**

- Systematic evaluation of compiled published study data illustrating factors influencing overall concordance of the original PGT‐A and reanalysis results.

## 1 INTRODUCTION

Preimplantation genetic testing for aneuploidy (PGT‐A) is a proven intervention in the treatment of infertility, with decreased clinical miscarriage risk, increased delivery rates from the first embryo transfer attempt, and reduced risk of multiple gestation without compromising success rates.^1^, ^2^, ^3^, ^4^, ^5^ However, the diagnostic predictive value of PGT‐A, when considering embryonic mosaicism,^6^, ^7^, ^8^ or segmental imbalance^9^, ^10^ remains controversial and has involved considerable resources to investigate.

Several commercially available PGT‐A methods involve predicting the presence of mosaicism from a single trophectoderm biopsy. These methods primarily rely upon classifying intermediate chromosome copy numbers as falling in a ‘mosaic range’.^7^ Mosaicism may primarily originate during embryonic mitotic cell division and chromosome nondisjunction, leading to daughter cells with different chromosomal constitution (trisomy, disomy, and/or monosomy).^6^ Contemporary methods of quantitation of chromosomes from a multicell sample (ie, trophectoderm) may identify mosaicism by observing intermediate copy number (ie, 2.6 instead of 3 in the case of mosaic trisomy) indicating a mixture of euploid and aneuploid cells. Different methods of classification have been proposed, including 20% to 80% (2.2‐2.8 mosaic range) and 30% to 70% (2.3‐2.7 mosaic range).^11^, ^12^

Several studies suggest that embryos classified as mosaic have reduced reproductive potential when compared to embryos classified as euploid.^13^, ^14^, ^15^, ^16^, ^17^ These clinical outcome data have largely been used as evidence for the validity of mosaicism diagnoses. Others have also argued that reduced success rates may be explained by false negative uniform aneuploidy predictions.^6^, ^7^, ^8^ That is, embryos classified as mosaic aneuploid may actually be uniform aneuploid in a large percentage of cases, leading the observed reduction in clinical success rates.^11^, ^15^ Conversely, many observations of successful outcomes following transfer of embryos classified as mosaic have also been made.^17^, ^18^ While some have argued that this is evidence of ‘self‐correction’,^19^ others have suggested that these embryos may have been misclassified as mosaic and were actually uniformly euploid.^20^

Another relatively new category of PGT‐A classification involves detection of de novo segmental imbalance. Detection limits (minimal size of segmental imbalances) vary by platform used, but generally consider the possibility of detecting imbalances that are 10 Mb or larger. Unlike embryos classified as ‘mosaic’, very little data exists regarding the clinical outcomes of transferring embryos with putative segmental imbalance. However, some studies have indicated that the primary origin of de novo segmental imbalance is mitotic, leading to a higher prevalence of mosaicism when segmental imbalance is observed.^21^, ^22^

To date, only two ‘nonselection’ studies to investigate the clinical predictive value of PGT‐A have been published.^2^, ^23^ This involves performing PGT‐A without using the information to select embryos for transfer, followed by unblinding PGT‐A results once actual clinical outcomes have been determined. Similar studies involving the transfer of embryos classified as mosaic or segmental aneuploid are currently underway.^24^, ^25^ While this study design is critical to understanding whether PGT‐A diagnoses are predictive of clinical outcomes,^26^ systematic evaluation of studies with reanalysis of PGT‐A diagnosed embryos may provide an alternative and meaningful strategy. For instance, clinical outcomes can be impacted by more than the genetic composition of the embryo, limiting its utility in addressing the predictive value of a single biopsy for the status of the remaining embryo. Comparing the concordance between multiple samples from the same embryo provides relevant information on the predictive value of the original test in terms of the overall diagnosis of an embryo, an important piece of data that cannot be obtained after a biopsied blastocyst is transferred to the uterus.

No published exhaustive compilation of studies reporting genetic testing on more than one sample from the same embryo is yet available. This is the first systematic review of published studies with data on multiple tests from human blastocysts. A detailed analysis of PGT‐A diagnosis concordance and distribution of chromosomal abnormality events amongst 1623 human blastocysts was performed.

## 2 METHODS

### 2.1 Study selection

Contemporary PGT‐A methods involve comprehensive screening of all 24 chromosomes and the use of blastocyst trophectoderm biopsy or spent blastocyst culture media samples. A general key word online search including PGT was performed, followed by referencing citations within each article and personal communications. Keywords used for online search included rebiopsy, PGT‐A, PGS, PGD, reanalysis, concordance, human embryo, and blastocyst. Published studies with PGT‐A results on more than one sample from the same blastocyst, and that involved PGT‐A for all 24 chromosomes (ie, single nucleotide polymorphism [SNP] microarray, quantitative real‐time PCR [qPCR], array comparative hybridization [aCGH], and next‐generation sequencing [NGS]) were identified and included for analysis (Table 1). Samples analyzed included trophectoderm biopsies and rebiopsies, the remaining whole embryo, and blastocyst outgrowth after in vitro extended culture. Studies reporting testing results in other samples such as blastomeres or products of conception were either partially or completely not included in this review. In addition, studies with incomplete data and/or that failed to provide missing data after request to the corresponding authors were excluded from the study. Data on cell‐free PGT‐A retests were not included in this review.

**TABLE 1:** Published studies included in this review with PGT‐A reanalysis data of human blastocysts

Study number	Study first author	Publication date	Embryos	Reference sample PGT‐A method	Reanalysis sample type	Retest PGT‐A method
1	Fragouli^27^	2008 November	10	CGH	ICM	CGH
2	Northrop^28^	2010 August	50	SNP array	TE/ICM	SNP array
3	Fragouli^29^	2011 February	19	CGH	TE	aCGH
4	Treff^30^	2012 April	71	SNP array	TE	qPCR
5	Capalbo^31^	2015 June	155	aCGH	TE	qPCR
6	Huang^32^	2016 June	30	aCGH	TE	NGS
7	Orvieto^33^	2016 June	8	NGS	TE/ICM	NGS
8	Huang^34^	2017 April	51	aCGH	TE/ICM	NGS
9	Zimmerman^35^	2018 January	31	qPCR	TE	NGS
10	Kuznyetsov^36^	2018 May	24	NGS	Whole blastocyst	NGS
11	Tsuiko^37^	2018 June	14	NGS	ICM	NGS
12	Popovic^38^	2018 July	58	NGS	TE/ICM	NGS
13	Chuang^39^	2018 December	29	NGS	TE/ICM	NGS
14	Victor^16^	2019 January	100	NGS	TE/ICM	NGS
15	Victor^40^	2019 February	8	NGS	TE/ICM	NGS
16	Popovic^41^	2019 April	45	NGS	Blastocyst outgrowth	NGS
17	Lawrenz^42^	2019 June	84	NGS	TE/ICM	NGS
18	Huang^43^	2019 July	50	NGS	Whole blastocyst	NGS
19	Treff^44^	2019 August	14	NGS	TE	SNP array
20	Munne^45^	2020 January	57	NGS	TE	NGS
21	Ou^46^	2020 January	63	NGS	Whole blastocyst	NGS
22	Sachdev^47^	2020 February	32	NGS	TE/ICM	NGS
23	Girardi^9^	2020 March	88	NGS	TE/ICM	NGS
24	Navratil^48^	2020 April	75	NGS	TE/Whole blastocyst	NGS
25	Rubio^49^	2020 May	64	NGS	ICM	NGS
26	Lin^50^	2020 June	41	NGS	ICM	NGS

Abbreviations: aCGH, array comparative hybridization; CGH, comparative genomic hybridization; ICM, inner cell mass; NGS, next‐generation sequencing; qPCR, quantitative real‐time PCR; PGT‐A, preimplantation genetic testing for aneuploidy; SNP, single nucleotide polymorphism; TE, trophectoderm.

### 2.2 Retest concordance analysis

Concordance analysis was performed by considering the original trophectoderm biopsy as the reference result. When the study did not classify one sample as the original test result, or the original test was a ‘no result’, a second trophectoderm biopsy was designated as the reference. Results were classified as (a) euploid (46,XX, 46,XY or 46,– without report of sex chromosomes), (b) full aneuploid (including segmental imbalance or polyploidy), or (c) mosaic aneuploid (any sample with at least one study‐specific mosaic range chromosomal aneuploidy designation, including segmental imbalance). Samples designated as chaotic, complex aneuploid, no result, involving PGT‐SR (structural rearrangements) or generated from parents with abnormal karyotypes were excluded from the concordance analysis. If more than one reanalysis of a blastocyst was performed, each comparison to the reference result was considered independently. PGT‐SR cases were used as a positive control for uniform aneuploidy, since the imbalances originate from known meiotic error.

Concordance values with the reference samples were obtained and compared across several subsets of studies, including those with and without (a) NGS in the original test result, (b) mosaicism classification in the original test result, or (c) segmental imbalance detection in the original test result, using a Fisher's exact test. A *P*‐value of less than .05 was considered significant.

### 2.3 Whole embryo concordance analysis

Based on all PGT‐A results available for each embryo, all blastocysts were further classified as uniform euploid (if all test results retrieved the same euploid karyotype), uniform aneuploid (if all test results retrieved an identical and abnormal karyotype), mosaic by discordance (if at least one karyotype differed from the rest from that same embryo), and mosaic by reciprocal (if at least one complimentary trisomy/monosomy event was observed). If a gain of a whole chromosome or a segment was observed in one sample and a loss of the same chromosome or segment was observed in another sample of the same blastocyst, these embryos were further categorized as segmental or whole chromosome reciprocal. If a reciprocal aneuploidy event involved one whole chromosome in one biopsy and one segment of the same chromosome in another biopsy, the embryo was classified as segmental reciprocal.

## 3 RESULTS

Thirty‐seven studies published in peer‐reviewed journals were retrieved after the search was performed. From these, one study performed a retest in leftover amplified DNA, six performed a retest in embryo spent culture media only, two involved testing products of conception, and two studies failed to provide necessary data for analysis. As a result, 26 published studies including 24‐chromosome PGT‐A and analysis of two or more blastocyst‐derived samples were identified (Table 1).^9^, ^16^, ^27^, ^28^, ^29^, ^30^, ^31^, ^32^, ^33^, ^34^, ^35^, ^36^, ^37^, ^38^, ^39^, ^40^, ^41^, ^42^, ^43^, ^44^, ^45^, ^46^, ^47^, ^48^, ^49^, ^50^ A total of 1271 embryos were included in this review. In addition, 147 embryos were from patients who had an abnormal karyotype were used for analysis of positive controls.

### 3.1 Positive controls

Data from a total of 147 embryos (310 comparisons) from five studies were obtained from cases in which either the patient or the partner had a chromosomal structural rearrangement or sex chromosome aneuploidy (Table S1). 93.5% of the reference samples were full aneuploid involving a chromosome present in the parental structural rearrangement, 5.8% were euploid and 2 (0.6%) reference samples were mosaic aneuploid for a chromosome involved in the abnormal parental karyotype. Euploidy concordance rate was 100%, whereas full aneuploidy concordance was 98.97% (97.01‐99.79 95% CI), where three retest samples were diagnosed as euploid. Of the two mosaic aneuploid reference samples, one showed a euploid result and the other one a full aneuploid result in the retest.

### 3.2 Retest concordance analysis

**FIGURE 1:**
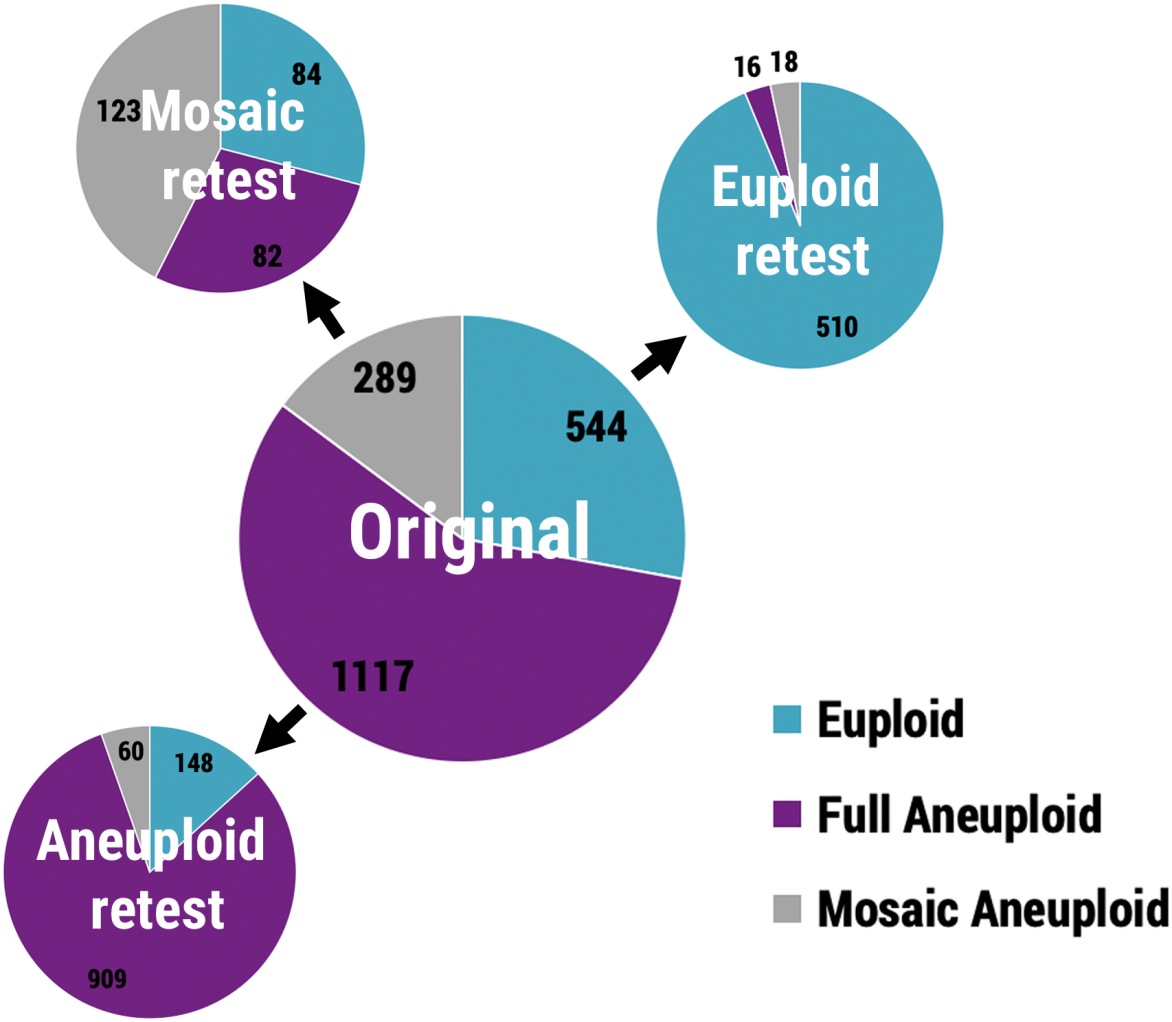
Distribution of blastocyst retest results for concordance analysis from a total of 1950 original reference results (trophectoderm biopsies). Numbers refer to independent comparisons [Colour figure can be viewed at wileyonlinelibrary.com]

**FIGURE 2:**
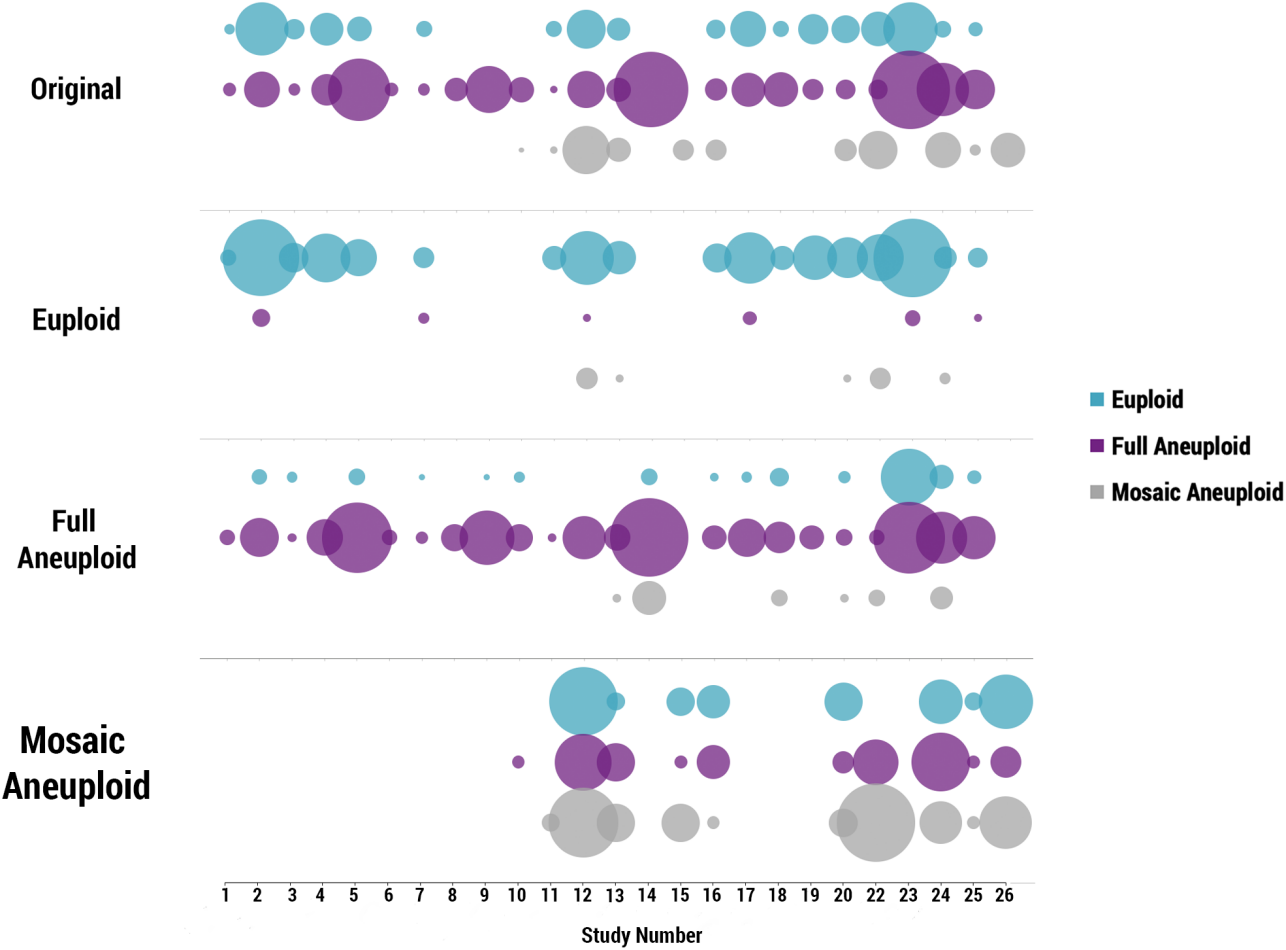
Reference sample and retest results distribution for each of the 25 published studies included in the concordance analysis. Upper plot ‘Original’ displays the diagnoses for each reference sample. Second plot ‘Euploid’ shows the distribution of retest results of embryos with an original euploid diagnosis. Same is shown for original ‘Full Aneuploid’ and ‘Mosaic Aneuploid’ diagnoses. Size of circles is proportionate to the sample size of each study, see Table S2. X axis refers to study number as described in Table 3 [Colour figure can be viewed at wileyonlinelibrary.com]

A total of 1124 embryos were analyzed leading to 1950 pairwise comparisons. The overall cohort included 544 euploid, 1117 full aneuploid, and 289 mosaic aneuploid reference samples. Total concordance rates of reanalysis results with the original PGT‐A results were 93.8% for euploidy, 81.4% for full aneuploidy, and 42.6% for mosaic aneuploidy (Table 2 and Figures 1 and 2), which were all significantly different (*P* < .05). Data compiled from each study for concordance analysis is available in Table S2.

**TABLE 2:** Blastocyst PGT‐A retest concordance analysis of all 1124 embryos and 1950 comparisons with a reference result

PGT‐A diagnosis	Concordant comparisons	Nonconcordant comparisons	Concordance with reference (%)	Concordance with reference range among studies (%)	
				Min.	Max.
Euploidy	510	34	93.75	77.78	100
Full aneuploidy	909	208	81.38	40	100
Mosaic aneuploidy	123	166	42.56	0	100

Abbreviation: PGT‐A, preimplantation genetic testing for aneuploidy.

A series of three subset analyses were performed (Table 3). The first subset analysis involved evaluation of concordance data from published studies using NGS in the original biopsy compared to studies not using NGS in the original biopsy (Table 4). Euploidy concordance was 92.2% with NGS and 97.1% without NGS (*P* = .0053). Full aneuploidy concordance was 75.9% with NGS and 94.8% without NGS (*P* < .00001). Concordance of mosaic aneuploidy was 42.6% with NGS, but unavailable from studies without NGS (mosaicism designations were not made).

**TABLE 3:** Study subset description

Study number	Study first author	PGT‐SR cases	NGS	Mosaic	Segmental
1	Fragouli^27^				✓
2	Northrop^28^				
3	Fragouli^29^				
4	Treff^30^				
5	Capalbo^31^				
6	Huang^32^	✓			✓
7	Orvieto^33^		✓		
8	Huang^34^	✓			✓
9	Zimmerman^35^				
10	Kuznyetsov^36^	✓	✓	✓	✓
11	Tsuiko^37^		✓	✓	
12	Popovic^38^		✓	✓	✓
13	Chuang^39^		✓	✓	✓
14	Victor^16^		✓		✓
15	Victor^40^		✓	✓	✓
16	Popovic^41^		✓	✓	✓
17	Lawrenz^42^		✓		✓
18	Huang^43^		✓		✓
19	Treff^44^		✓		
20	Munne^45^		✓	✓	✓
21	Ou^46^	✓			
22	Sachdev^47^		✓	✓	✓
23	Girardi^9^	✓	✓		✓
24	Navratil^48^		✓	✓	✓
25	Rubio^49^		✓	✓	✓
26	Lin^50^		✓	✓	✓

*Note:* Cells highlighted in light blue indicate in which study subset each study was categorized.

Abbreviations: NGS, next‐generation sequencing; PGT‐SR, preimplantation genetic testing for structural rearrangements.

**TABLE 4:** Blastocyst PGT‐A retest concordance analysis for the NGS and no NGS study subsets

PGT‐A diagnosis	NGS			No NGS		
	Euploidy	Full aneuploidy	Mosaic aneuploidy	Euploidy	Full aneuploidy	Mosaic aneuploidy
Concordant comparisons	342	600	123	168	309	N/A
Nonconcordant comparisons	29	191	166	5	17	N/A
Concordance with reference (%)	92.18	75.85	42.56	97.11	94.79	N/A
Concordance range among studies (%)	77.78‐100	46.15‐100	0‐100	94.85‐100	40‐100	N/A
*P‐*value				.0053	<.00001	N/A

*Note: P*‐values were obtained after computing a Fisher's exact test comparing PGT‐A diagnosis concordances between the two study subsets.

Abbreviations: NGS, next‐generation sequencing; PGT‐A, preimplantation genetic testing for aneuploidy.

The second subset analysis involved evaluation of concordance data from published studies incorporating prediction of segmental imbalance to studies not incorporating prediction of segmental imbalance in the original test result (Table 5). Euploidy concordance was 91.7% in studies incorporating segmental imbalance prediction and 96.8% in studies not incorporating segmental imbalance prediction (*P* = .018). Full aneuploidy concordance was 76.3% in studies incorporating segmental imbalance prediction and 94.3% in studies not incorporating segmental imbalance prediction (*P* = .00001). Concordance of mosaic aneuploidy was 42.2% in studies incorporating segmental imbalance prediction, but generally unavailable from studies not incorporating segmental imbalance prediction (n = 2 available comparisons).

**TABLE 5:** Blastocyst PGT‐A retest concordance analysis for the segmental aneuploidy study subsets

PGT‐A diagnosis	Segmental			No segmental		
	Euploidy	Full aneuploidy	Mosaic aneuploidy	Euploidy	Full aneuploidy	Mosaic aneuploidy
Concordant comparisons	299	610	121	211	299	2
Nonconcordant comparisons	27	190	166	7	18	0
Concordance with reference (%)	91.72	76.25	42.16	96.79	94.32	100.00
Concordance range among studies (%)	80‐100	46.15‐92.50	25‐74.51	77.78–100	40‐100	100
*P‐*value				.0182	<.00001	.1803

*Note: P*‐values were obtained after computing a Fisher's exact test comparing PGT‐A diagnosis concordances between the two study subsets.

Abbreviation: PGT‐A, preimplantation genetic testing for aneuploidy.

The third subset analysis involved evaluation of concordance data from published studies incorporating prediction of mosaicism to studies not incorporating prediction of mosaicism in the reference sample (Table 6). Euploidy concordance was 88.8% in studies incorporating mosaicism prediction and 96.1% in studies not incorporating mosaicism prediction (*P* = .002). Full aneuploidy concordance was 81.3% in studies incorporating mosaicism prediction and 84.4% in studies not incorporating mosaicism prediction (*P* = .237). Concordance of mosaic aneuploidy was 42.6% in studies incorporating mosaicism prediction, but unavailable from studies not incorporating mosaicism prediction (mosaicism designations were not made).

**TABLE 6:** Blastocyst PGT‐A retest concordance analysis for the mosaicism study subsets

PGT‐A diagnosis	Mosaics			No mosaics		
	Euploidy	Full aneuploidy	Mosaic aneuploidy	Euploidy	Full aneuploidy	Mosaic aneuploidy
Concordant comparisons	158	231	123	352	857	N/A
Nonconcordant comparisons	20	53	166	14	158	N/A
Concordance with reference (%)	88.76	81.34	42.56	96.17	84.43	N/A
Concordance range among studies (%)	80–100	46.15–100	0–100	77.78‐100	40–100	N/A
*P‐*value				.002	.237	N/A

*Note: P*‐values were obtained after computing a Fisher's exact test comparing PGT‐A diagnosis concordances between the two study subsets.

Abbreviation: PGT‐A, preimplantation genetic testing for aneuploidy.

### 3.3 Whole embryo concordance analysis

Although a chromosomal mosaicism diagnosis is often provided after analysis of a single trophectoderm biopsy, more rigorous and stringent criteria for this categorization involve the evaluation of more than one biopsy from the same embryo.^6^ As a result, the 1124 embryos were further classified as either nonmosaic (uniform euploid or uniform aneuploid), or mosaic (when at least one retest result differed from the reference sample result). 24.4% of embryos were uniform euploid and 39.9% were uniform aneuploid, leaving a 35.7% of embryos categorized as mosaic. Furthermore, from the 402 mosaic embryos, 360 (32.0% of the total) were classified as mosaic solely by discordance, and 42 (3.7%) as mosaic by reciprocal (when at least one reciprocal aneuploidy event was observed). Moreover, nearly half of the embryos categorized as mosaic by reciprocal (24/42) involved a segmental reciprocal aneuploidy event (Table 7).

**TABLE 7:** Whole embryo concordance analysis

Category	All studies		No segmentals reported		Segmentals reported		Fisher's exact test (*P*)
	Embryos (n)	Rate (%)	Embryos (n)	Rate (%)	Embryos (n)	Rate (%)	
Uniform euploid	274	24.4	123	34.0	151	19.8	<.00001
Full concordance uniform aneuploid	448	39.9	184	50.8	264	34.6	<.00001
Mosaic by discordance	360	32.0	55	15.2	305	40.0	<.00001
Mosaic by reciprocal	42	3.7	0	0.0	42	5.5	<.00001
Mosaic by segmental reciprocal	24	2.1	—	—	24	3.1	—
Total	1124		362		762		

Finally, when only embryos from studies reporting segmental imbalances in the original test were analyzed, 45.5% were classified as mosaic in contrast to 15.2% of embryos from studies not reporting segmental imbalances (*P* < .00001). Embryos classified as mosaic by reciprocal events were only observed when segmental imbalances were reported (Table 7).

## 4 DISCUSSION

This study provides the first systematic review of published data involving reanalysis of blastocyst PGT‐A diagnoses. Given the heterogeneity of data included in this review, the cohort of human blastocyst evaluated in the present study may not be representative of embryos generated by routine IVF in the clinical setting. This selection bias warrants caution when considering the absolute frequency of abnormalities observed. However, relative quantitation of concordance rates from this study are relevant to the predictive value of a single biopsy for the remaining embryo.

One important component of the present study was to demonstrate methodological validity by performing analysis on a set of positive controls. Aneuploidy of meiotic origin is expected to give consistent (uniform) results from reanalysis. Indeed, this was observed when evaluating studies of embryos from patients carrying a structural rearrangement (99%‐100% concordance). This illustrates that initial PGT‐A results are capable of accurately predicting the chromosomal status of the remaining embryo. Nonetheless, two embryos were reported as mosaic aneuploids for a chromosome involved in the parental chromosomal rearrangement, which highlights the importance of individual laboratory criteria to diagnose mosaicism.

The introduction of new methods of PGT‐A, namely NGS‐based testing and prediction of mosaicism and de novo segmental imbalance, was subsequently evaluated and demonstrated significant discordance upon reanalysis. Of particular interest is the interpretation of mosaicism. Reanalysis of embryos predicted to have mosaic range aneuploidy demonstrated poor predictive value for the remaining embryo. One perspective on these results is that this is completely expected. That is, if the embryo is truly mosaic, remaining portions of the embryo should vary from completely euploid in some, to mosaic or completely aneuploid in others. Another interpretation of these data is that a prediction of mosaicism from a single biopsy is inaccurate. In either case, current methods of mosaicism diagnoses from a single embryo biopsy are poorly predictive of the remaining embryo.

Current clinical methods of classifying embryos as mosaic involves evaluating intermediate copy numbers with varying thresholds using a single biopsy. Some laboratories may use 20% to 80% (liberal criteria), while others may use 30% to 70% (conservative criteria), with dramatic differences in the predicted prevalence of mosaicism.^6^ Another type of classification involves characterizing multiple biopsies from the same embryo. Again, the criteria used can have a dramatic impact on the prevalence observed. For example, considering any embryo in this dataset with discordant karyotype (liberal criteria) results in predicting a 32% mosaicism rate. In contrast, considering only embryos with reciprocal aneuploidy (conservative criteria) results in predicting a 3.7% mosaicism rate.

At minimum, the results presented here warrant the consideration of alternative explanations for clinical outcomes following transfer of putative ‘mosaic’ embryos. In cases where an embryo is classified as ‘high range’ mosaic, it may be possible that they were actually uniformly aneuploid. Transferring these embryos and observing reduced reproductive potential cannot rule out this interpretation without direct reanalysis of the remaining embryo. In the present study, approximately one‐third of mosaic embryo was found to possess a full aneuploidy upon reanalysis. This is consistent with observations made by Handyside et al, published in this issue of Prenatal Diagnosis, which indicate that approximately one‐third of embryos with chromosomes designated as mosaic by NGS copy number analysis were actually meiotic in origin when further evaluated.

Conversely, in cases where an embryo is classified as ‘low range’ mosaic, it may be possible that they were actually uniformly euploid. Transferring these embryos and observing healthy deliveries cannot rule out this interpretation without direct reanalysis of the remaining embryo. In the present study, approximately one‐third of mosaic embryos were found to possess uniform euploidy upon reanalysis. This is consistent with clinical outcomes, where approximately one‐third lead to healthy deliveries.^17^

Blastocyst mosaicism is undoubtedly a real phenomenon. While the use of intermediate copy numbers from a single biopsy alone may be insufficient to accurately predict the remaining embryo, there may be several useful innovations to consider. Given that mitotic nondisjunction is a commonly observed mechanism leading to mosaicism,^51^, ^52^ methods that can distinguish meiotic and mitotic origins of aneuploidy (from a single biopsy) may improve specificity of mosaicism predictions. In addition, given that mosaicism originates during post‐zygotic cell division events and is commonly associated with micronuclei, time‐lapse morphokinetics may also be instrumental in improving specificity.^53^

Significantly different discordance rates were also observed when evaluating the use of segmental imbalance predictions. Very little data on the clinical predictive value of segmental imbalance has been developed. Given the challenges already faced with regard to interpretation of clinical outcomes of putative mosaic embryos, and based on this review, more careful consideration of methodologies used to predict segmental imbalances is warranted. As with predicting mosaicism from intermediate copy numbers alone, determining cell division origins of segmental abnormalities may also improve the specificity of their diagnoses from a single biopsy.

These findings stress the burden on clinical management of embryos diagnosed as mosaic or with segmental imbalances. Although more rigorous and well‐designed studies evaluating the predictive value of these diagnoses are imperative to provide an answer to this issue, genetic testing laboratories should reconsider more rigorous validation and criteria for improving the accuracy of predictions. Chromosome copy number alone is insufficient to predict a true mosaic result. Despite the apparent rush to incorporate mosaicism diagnoses into clinical use, several studies indicate reduced utilization of mosaicism reporting. In addition to the uncertainty surrounding copy number based prediction of mosaicism, the studies included in this review also illustrate enormous variability in data reporting. For example, it was impractical to separately analyze mosaicism predictions based on study‐specific copy number thresholds.

PGT‐A methodological development has largely been driven by decreasing genetics laboratory costs, increasing throughput capacity, and access to commercially available testing kits.^54^ While NGS is currently the preferred technology for PGT‐A, concordance rates differ significantly from several other testing platforms (Table 4). Based upon the results presented here, it is likely that increased discordance rates are a result of including mosaicism and segmental imbalance predictions. These approaches may ultimately lead to inaccuracy of embryo diagnoses, and, as is currently the case, present an unnecessary clinical conundrum for patients and physicians to manage.

## 5 CONCLUSIONS

This systematic review of published studies illustrates the need for caution when developing new capabilities from PGT‐A methods of data analysis. While mosaicism and segmental imbalances exist, estimating its frequency in the human blastocyst is dramatically affected by criteria used to predict its presence from single biopsy. Clinical outcomes alone are insufficient to validate new methods of prediction. Direct evaluation of the predictive value for the remaining embryo clearly demonstrates insufficient concordance from many testing methodologies and should be acknowledged when considering the putative impact on reproductive potential.

## CONFLICT OF INTEREST

Diego Marin, Jia Xu, and Nathan R. Treff are employees or shareholders of Genomic Prediction.

## Supporting information


**Table S1** Blastocysyt retest concordance data for all studies with embryos generated from patients/partners with abnormal karyotypes.

**Table S2** Blastocysyt retest concordance data for all studies included in the analysis.

## Data Availability

Data compiled from each study is available in Supplementary files.
